# Genetic diversity analysis for wild and cultivated accessions of *Cymbopogon citratus* (D.C.) Stapf using phytochemical and molecular markers

**DOI:** 10.7717/peerj.13505

**Published:** 2022-06-29

**Authors:** Bushra Shamsheer, Nadia Riaz, Zubaida Yousaf, Sajjad Hyder, Arusa Aftab, Rashid Iqbal, Muhammad Habib ur Rahman, Ibrahim Al-Ashkar, Khalid F. Almutairi, Ayman El Sabagh

**Affiliations:** 1 Department of Botany, Lahore College for Women University, Lahore, Pakistan; 2 Department of Botany, Government College Women University, Sialkot, Pakistan; 3 Department of Agronomy, Faculty of Agriculture and Environment, The Islamia University of Bhawalpur, Bahawalpur, Pakistan; 4 Crop Science Institute of Crop Science and Resource Conservation (INRES), University of Bonn, Bonn, Germany; 5 Department of Plant Production, College of Food and Agriculture, King Saud University, Riyadh, Saudi Arabia; 6 Department of Agronomy, Faculty of Agriculture, Kafrelsheikh University, Kafr El-Shaikh, Egypt

**Keywords:** *Cymbopogon citratus*, Cultivated, Genetic diversity, Molecular markers, Medicinal importance, Oil yielding, Phytochemicals, Wild

## Abstract

**Background:**

Genetic diversity is being lost because of increasing urbanization and decreasing cultivation land, which leads to the abrupt use of wild resources of medicinally aromatic plants (MAPs). *Cymbopogon citratus* is a morphologically diverse MAP that is largely exploited in the food, cosmetics, and pharmaceutical industries. However, the intraspecific phytochemical and molecular diversity of *C. citratus* has yet to be explored.

**Methodology:**

The germplasm was obtained from four different countries representing Pakistan, India, Bangladesh, and the United States. Oil extraction was performed by hydro distillation, and metabolic profiles of different accessions were generated by GC–MS. Seventeen functional molecular markers based on three genes encoding cytochrome P450, uridyl diphosphate glycosyltransferase and the 5S rRNA gene family were used to explore genetic diversity. Principal component analysis (PCA) and heatmaps were constructed using R software with the help of the gg-plot R package v1.0.5 for data validation.

**Results:**

Among the 208 identified metabolites, citral was maximal, with a phytochemical contribution (1.92–27.73%), α-pinene (0.82–15.57%), verbenol (0.24–22.84%), neral (0.23–21.31%) and geranial acetate (0.43–15.65%). In the majority of accessions, citral was the dominant component. The highest concentration of citral was detected in 384541 (27.74%), 384527 (27.52%) belonging to Pakistan and one USA-based accession 38456 (27.71%). Region-specific grouping revealed a relationship between genetic diversity and geographical location. Pakistani accessions 384518, 38452, and 384544 genetically and 384535, 384518, and 384510 were phytochemically diverse.

**Conclusion:**

The genetic diversity was more pronounced in cultivated accessions than in wild accessions. Moreover, it was observed that phytochemical diversity correlated with the altitude and temperature of the region.

## Introduction

Essential oils (EOs) are complex mixtures of secondary metabolites extensively used in cosmetics, pharmaceutics, and food additive industries (Ramirez et al., 2021). Currently, the global demand has reached up to US $27 billion. Europe has a major share of the production of essential oil, followed by Asia Pacific regions and North America (Ridder, 2021). The major EO-yielding crops are Mentha, Lavender, Eucalyptus, *etc*. To meet the increasing demand, it is necessary not only to increase the yield of existing crops but also to find new potential resources for EO production (Verdeguer et al., 2020). *Cymbopogon citratus* (D.C.) Stapf, could be a significant source of essential oil (Silva et al., 2010). It is native to Asia, where tropical and subtropical conditions are favorable for the cultivation of this species. South Asian countries such as Pakistan and India could be good reservoirs for cultivated and wild resources of *C. citratus* (Anal, 2014). However, the production of EO-yielding crops, including *C. citratus*, is not enough to meet the required demand in this region. Therefore, during 2020, Pakistan made the import of EOs Singapore with a net worth of US $52.42 million (Rocha & Melo, 2011).

The essential oil of *C. citratus* serves as a rich source of citral, geranial, neral, α-pinene, verbenone, and limonene (Plata-Rueda et al., 2020). Among all metabolites, citral is an important component. Citral is a natural flavoring agent used in food, beverages, pharmaceuticals, cosmetics and acts as a raw material for the production of beta-carotene and vitamin A (Abdelrahman et al., 2020). Moreover, citral has a wide range of medicinal uses, such as in the treatment of malaria, diabetes, pneumonia, anxiety, flu, and fever (Tchoumbougnang et al., 2005). Moreover, it possesses anticancer, antioxidant, antifungal, and antibacterial properties (Mukarram et al., 2021). Despite its multiple applications, the stability of citral is limited to its environmental conditions and geographical origin (Chat et al., 2019).

The environment plays a key role in determining the phytochemical diversity of plants (Kumar et al., 2017). The EO composition of *C. citratus* is influenced by many endogenous (genetic variability, plant part, maturity stage) and exogenous (temperature, humidity, soil type) factors. These endogenous and exogenous factors are fully recognized for some aromatic and medicinal plants but are limited for this plant species (Moghaddam & Mehdizadeh, 2017). Many medicinal and aromatic plants (MAPs) are at risk of extinction because of their extensive use for the manufacturing of herbal medicines as well as loss of habitat (Gurib-Fakim et al., 2002). Hence, in the present scenario, the conservation of plants ensures the maintenance of their sustainable utilization for food and medicine. The production of MAPs *via* cultivation can reduce pressure on wild resources and can maintain consistency in production (Noorhosseini, Fallahi & Damalas, 2019). Moreover, the exploration of genetic diversity is a key step for the improvement of the crop. This provides an opportunity to plant breeders for the development of new cultivars with desirable traits.

The common methods for essential oil extraction are hydrodistillation, steam distillation, and microwave distillation (Flamini et al., 2007). For the selection of the best germplasm, the association of phytochemical and molecular markers is of prime importance. To promote cultivation, the best genotype and high-yielding parental lines should be selected by determining the genetic diversity of *C. citratus*. The diversity of these aromatic species can be obtained through chemotaxonomic markers. RAPD, SSR, and functional molecular markers have been used previously for the determination of genetic diversity in different crops (Khanuja et al., 2005). However, the diversity of *C. citratus* within the species has yet to be explored.

The present study aimed to evaluate the phytochemical and molecular diversity among the accessions collected from different geographical areas of Pakistan, India, Bangladesh, and the United States to bring them into cultivation.

## Material and Methods

### Plant material

A panel of forty accessions of *C. citratus*, including twenty cultivated and twenty wild accessions, were collected spanning South Asia (Pakistan, India, Bangladesh) and America to explore the molecular and phytochemical diversity. The collected germplasm was submitted to the GenBank of the Institute of Agro-Biotechnology and Genetic Resources (IABGR). The specimens were submitted to the National Agriculture Research Centre (NARC), Islamabad, Pakistan, for voucher numbers and tiller multiplication for future use (Table 1).

**Table 1:** Geographical location and physical factors of all accessions of *C. citratus*.

Accession no	Place of collection	Source	Altitude (m)	Latitude	Temperature (°C)	Rainfall (mm)
38452	Alipur Chatha	Wild	193	32.2654°N–73.8125°E	23.9	578
38453	Gujranwala (Kotshera)	Wild	231	32.1877°N–72.1945°E	23.9	578
38454	Dhaka (Bangladash)	Cultivated	4	23.8103°N–90.4125°E	25.9	2,022
38455	Sheikhupura	Cultivated	236	31.7167°N–73.9850°E	24.1	476
38456	USA (Virgin Island)	Cultivated	474	18.3358°N–64.8963°W	25.2	1,200
38457	Khairpur	Wild	61	26.8822°N–69.0970°E	26.9	99
38459	Phalia	Cultivated	205	32.4327°N–73.5771°E	24.0	530
384510	Kunjah	Wild	233	22.2587°N–71.1924°E	23.8	746
384511	Lahore	Cultivated	217	31.5204°N–74.3587°E	24.1	607
384512	Amritsar (India)	Cultivated	232	31.6340°N–74.872°E	23.4	703
384513	Pattoki	Wild	186	31.0249°N–73.8479°E	24.3	340
384514	Pattoki	Cultivated	186	31.0249°N–73.8479°E	24.3	340
384515	Halla	Wild	178	31.1199°N–73.7272°E	24.3	340
384516	Manawala (Faisalabad)	Wild	183	31.4504°N–73.1350°E	24.2	346
384518	Faisalabad	Cultivated	183	31.4504°N–73.1350°E	24.2	346
384519	Bani Gala (Islamabad)	Wild	540	33.6844°N–73.0479°E	21.3	941
384520	Islamabad	Cultivated	540	33.6844°N–73.0479°E	21.3	941
384521	Bahawalpur	Wild	214	29.3544°N–71.6911°E	23.8	187
384522	Sargodha	Cultivated	190	32.0740°N–72.6861°E	23.8	410
384523	Pindi	Wild	508	33.5651°N–73.0169°E	21.5	941
384524	Peshawar	Cultivated	331	34.0151°N–71.5249°E	22.7	384
384526	Multan	Cultivated	122	30.1575°N–71.5249°E	25.6	175
384527	Hafizaabad	Cultivated	200	32.0712°N–73.6895°E	24.1	437
384528	Hafizaabad	Wild	200	32.0712°N–73.6895°E	24.1	437
384529	Karachi	Cultivated	8	24.8607°N–67.0011°E	25.9	194
384530	Karachi	Wild	8	24.8607°N–67.0011°E	25.9	194
384531	Karachi	Wild	8	24.8607°N–67.0011°E	25.9	194
384532	Phull (Karachi)	Cultivated	8	24.8607°N–67.0011°E	25.9	194
384533	Shakarghar	Wild	268	32.2572°N–75.1604°E	26.2	722
384534	Kashmir	Cultivated	2,097	33.9259°N–73.7810°E	21.9	976
384535	Kashmir	Cultivated	2,097	33.9259°N–73.7810°E	21.9	976
384536	Bahawalnagar	Wild	163	30.0025°N–73.2412°E	25.1	204
384538	Kamoke	Cultivated	201	31.9765°N–74.2220°E	23.9	573
384540	Jhelum	Wild	234	32.9425°N–73.7257°E	23.6	842
384541	Daska	Cultivated	217	32.3363°N–74.3675°E	23.8	652
384542	Daska	Wild	217	32.3363°N–74.3675°E	23.8	652
384543	Dera Ismail khan	Wild	165	31.8626°N–70.9019°E	24.5	249
384544	Dera Ismail khan	Cultivated	165	31.8626°N–70.9019°E	24.5	249
384545	Pindi	Wild	508	33.5651°N–73.0169°E	21.5	941
384550	Bannu	Wild	327	32.9910°N–70.6455°E	25.6	249

### Extraction of essential oil from *C. citratus*

Mature leaves of each accession were collected and partially dried at room temperature for 72 h. The partially dried leaves of *C. citratus* (500 g) were subjected to hydrodistillation in 800 mL water in a clevenger-type apparatus for 4 h (Bagheri, Manap & Solati, 2014). The essential oil yield was calculated as the ratio between the volume of oil obtained and the weight of partially dried leaves used for oil extraction (Bistgani et al., 2018). The essential oil was collected in a clean vial, dried over sodium anhydrous sulfate and finally stored at 4 °C before being subjected to gas chromatography–mass spectrometry (GC–MS) analysis (Bagheri, Manap & Solati, 2014).

### GC–MS analysis

Different phytochemicals present in *C. citratus* were identified by GC–MS analysis following the procedure of Ranitha et al. (2014). GC–MS was performed using a GC TRACE-1300 chromatograph coupled with mass spectrometry, single quadrupole, and auto sampler (AI-1310; Thermo Scientific, Waltham, MA, USA). A Capillary column of TR-35 MS GC Column 30 mx, 25 mm IDx, 25 µm was used. The injector temperature was set at 280 °C, and the column temperature was initially maintained at 50 °C for 5 min and then programmed at 3 °C/min to 240 °C and 5 °C/min to 300 holds for 3 min. The initial sample delay time was 3.5 min. The transfer line temperature was set at 300 °C, while the ion source temperature was 250 °C. Helium (He) (99.99%) was used as the carrier gas with a linear gas flow of 1.5 mL/min through split-less injection. The injected volume was kept at 1 μL, and mass spectra were taken at 70 eV. Mass spectra identified different compounds of the oil compared with those of the computer library and based on their retention time and probability.

### Essential oil component identification

The essential oil components were identified by comparing GC retention indices (RIs) on polar and apolar columns, resolute relative to the RT of a sequence of n-alkanes with lined interpolation with those of reliable compounds and already published data (McLafferty & Stauffer, 1989). The computer mass spectra were also matched with the National Institute of Standards and Technology (NIST) mass spectral library. For the identification of citral and its components, citral standard (SIGMA-ALORICH) was injected into the GC–MS column under the same conditions for the test samples. The quantification of the components was accomplished by rendering the areas of the chromatographic peaks.

### Genetic diversity based on functional molecular markers

Genomic DNA was extracted from 2 g leaf samples of *C. citratus* using the CTAB method (Sánchez, Remarchuk & Ubayasena, 2006). Seventeen primers were selected from the literature based on the genes encoding cytochrome P450, uridyl diphosphate glycosyltransferase and the 5S rRNA gene family. These genes were selected based on their involvement in the metabolic pathways for secondary metabolites as reported in the literature.

### DNA amplification by polymerase chain reaction

Polymerase chain reaction (PCR) was performed by following the methodology of Luro et al. (2008). Bio–Rad T-100 thermal cycler (Bio–Rad, Hercules, CA, USA) in a final volume of 22 µL containing 5 µL template DNA, 1 µL of forward and reverse primer, 10 µL of 2X master mix (Wizbio) and 5 µL of nuclease free water. PCR conditions were kept as follows: denaturation at 94 °C for 5 min, followed by 39 repeats of 1 min at 94 °C, 1 min at 55–60 °C (depending on the melting temperature of the primers), 45 s at 72 °C, and the final extension for 5 min at 72 °C. Amplified PCR products were separated on a 2% high-resolution metaphor agarose gel (Cat. 50181; Lonza, Morristown, NJ, USA), prepared in TAE buffer. For this, 5 µL of PCR product was mixed with 1 µL of loading dye. containing 95% formamide, 0.25% bromophenol blue, 0.25% xylene cyanol, and 10 mM EDTA. Separated amplified DNA fragments were visualized on a UV-illuminator. The presence or absence of the band was scored 0 and 1, respectively. The polymorphism information content (PIC) value was calculated by following the methodology of Tams, Melchinger & Bauer (2005).

### Data analysis

Genetic relationships among different accessions were analyzed using DARwin software v6 (Perrier & Jacquemoud-Collet, 2006). Factorial analysis based on the simple matching similarity index was performed. The presence or absence of a band was scored as 1 and 0, respectively, for each of the samples. The polymorphic bands were included in the data analysis. Phytochemical data were analyzed using R studio software (version 1.0.5). Principal component analysis (PCA) and heatmaps were constructed to determine the relationship of wild and cultivated accessions based on phytochemical and genetic markers (Kassambara & Mundt, 2017).

## Results

### Composition of volatile compounds in *C. citratus*

**Figure 1:**
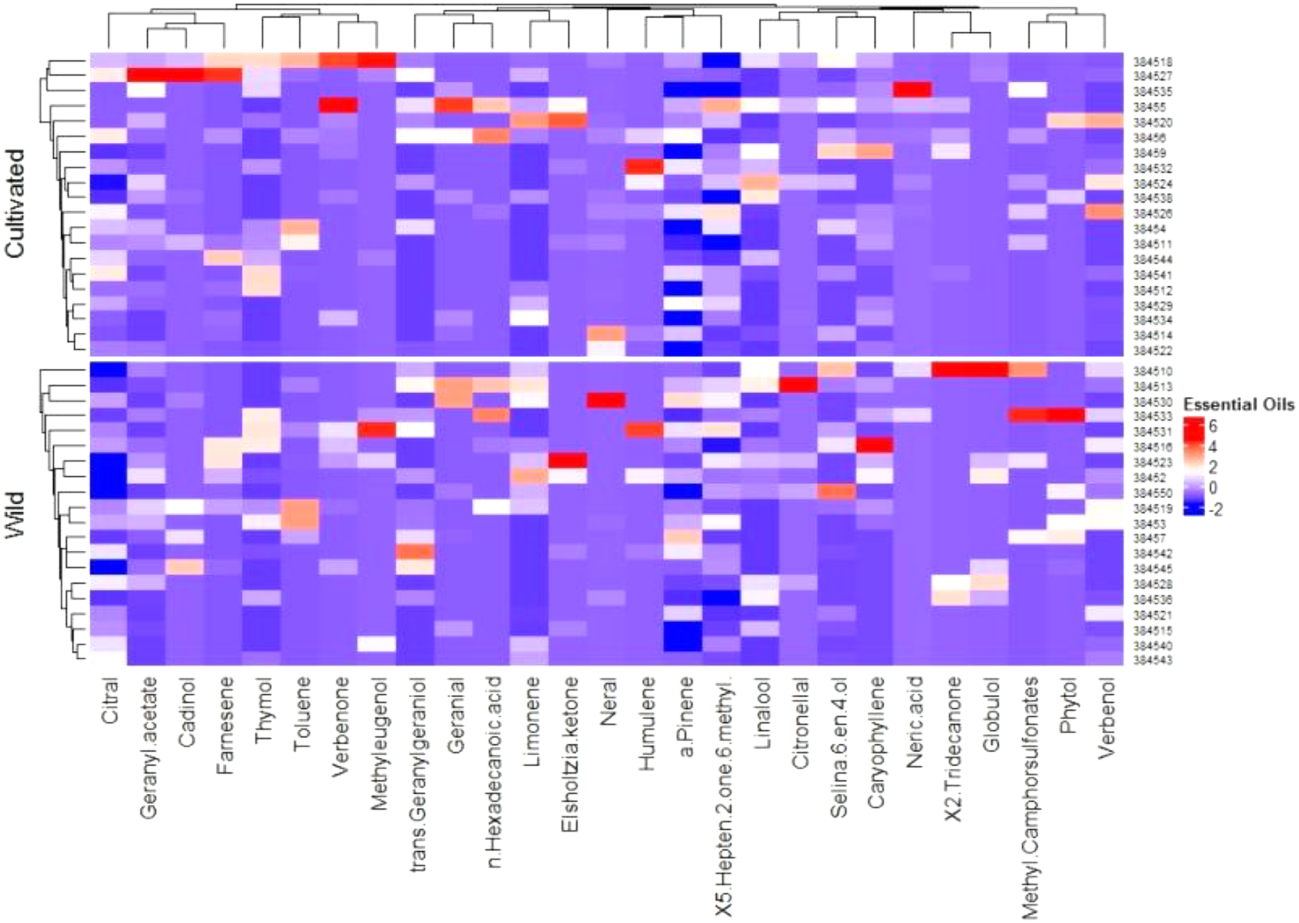
Heat map of *C. citratus* chemical diversity and the relationship based on standardized values for the proportions of EOs components between 20 cultivars (upper) and 20 wild (below).

The identified 208 compounds (https://pubchem.ncbi.nlm.nih.gov) constituted 94.6% to 99.8% of the total composition of *C. citratus* essential oils. Twenty-seven compounds with a proportion higher than 1% in at least one accession were observed (Table 2). To represent the possible imprint of the phytochemical profile in relation to the environment, a heatmap represented the arrangement of accessions into different groups (Fig. 1). In the cultivated accessions, the most striking phytochemical markers were geranyl acetate (15.96%), farnesene (1.65%), and cadinol (2.56%) for accession 384527 (Hafizabad) and verbenone (13.2% and 14.37%) for accessions 384518 (Faisalabad) and 38455 (Shiekupura). The accession 384535 (Karachi) showed a high proportion of neric acid (10.37%). The geranial concentration was 5.04% in accession 38455 (Shiekupura). In wild accessions, the maximum concentration of trans-geranylgeriniol (2.34%) was found in accession 384542 (Daska), citronellal (6.46%) in accession 384513 (Pattoki), and neral (21.31%) in accession 384530 (Karachi). Some compounds were detected at almost the same concentrations in both cultivated and wild accessions, such as methyleugenol in accessions 384518 (11.91%) and 384530 (10.35%). Humelene was detected at almost the same concentrations of 1.12% and 1.08% in accessions 384530 and 384531, respectively. The limonene concentrations were 5.67% and 4.37% in accessions 38452 and 384513, respectively. β-Caryophyllene and farnesene represented the sesquiterpenes. β-Caryophyllene was found in a higher proportion (7.29%) in wild accession 384516 (Faisalabad), while farnesene (1.65%) was found in cultivated accession 384527 (Hafizabad). Alcohols, including 3,6-octadien-1-ol, 3,7-dimethyl, cadinol, trans-geranylgeraniol, and linalool, were detected in both wild and cultivated accessions at varying concentrations. Among aldehydes, citral was dominant in most of the accessions, while neral geranial was not detected in any accession. Verbenol ketone was the most abundant ketone in the cultivated accessions, while Elsholtzia ketone was detected only in the wild accessions.

**Table 2:** Concentration of the abundant volatile compound in 40 accessions of *C. citratus*.

Sr. No	RT	Compounds name	% Peak area range	Molecular formula	Molecular weight g/mol
1	4.15	Toluene	8.36 ± 0.07	C_7_H_8_	92.14
2	12.15	α-Pinene	15.57 ± 0.82	C_10_H_16_	136.23
3	14.41	5-Hepten-2-one, 6-methyl	5.4 ± 0.68	C_8_H_14_O	126.2
4	22.03	Citronellal	6.46 ± 0.91	C_10_H_18_O	154.25
5	23.10	Limonene oxide	5.67 ± 0.08	C_10_H_16_O	152.23
6	27.57	Verbenol	22.84 ± 0.24	C_10_H_16_O	152.23
7	28.49	Geranial	5.04 ± 0.08	C_10_H_16_O	152.23
8	29.76	Citral	27.73 ± 1.92	C_10_H_16_O	152.23
9	30.44	Neral	21.31 ± 0.23	C_10_H_16_O	152.23
10	30.84	Linalool	5.65 ± 0.13	C_10_H_18_O	154.25
12	30.98	Thymol	1.08 ± 0.09	C_10_H_14_O	150.22
13	32.12	Neric acid	10.93 ± 0.43	C_10_H_16_O_2_	168.23
14	33.07	Geranyl acetate	15.65 ± 0.43	C_12_H_20_O_2_	196.29
15	34.17	β-Farnesene	1.65 ± 0.07	C_15_H_24_	204.35
16	35.23	Humelene	1.08 ± 0.06	C_15_H_24_	204.35
17	36.67	Methyleugenol	11.19 ± 0.65	C_11_H_14_O_2_	178.23
18	36.72	2-Tridecanone	3.17 ± 0.09	C_13_H_26_O	198.34
19	37.14	Verbenone	14.37 ± 0.84	C_10_H_14_O	150.22
20	41.61	β-Caryophyllene	7.29 ± 0.45	C_15_H_24_	204.35
21	43.23	Selina-6-en-4-ol	2.34 ± 0.05	C_15_H_26_O	222.37
23	42.41	Globulol	5.34 − 0.23	C_15_H_26_O	222.37
22	44.50	Cadinol	2.56 ± 0.12	C_15_H_26_O	222.37
23	53.90	n-Hexadecanoic acid	0.82 ± 0.06	C_16_H_32_O_2_	256.42
24	58.04	Phytol	3.25 ± 0.08	C_20_H_40_O	296.5
25	59.11	TransGeranylgeraniol	2.34 ± 0.42	C_20_H_34_O	290.5
26	61.70	Methyl-Camphorsulfonates	4.73 ± 1.02	C11H_18_O_4_S	246.33
27	63.98	Elsholtzia ketone	1.19 ± 0.05	C_10_H_14_O_2_	166.22

**Figure 2:**
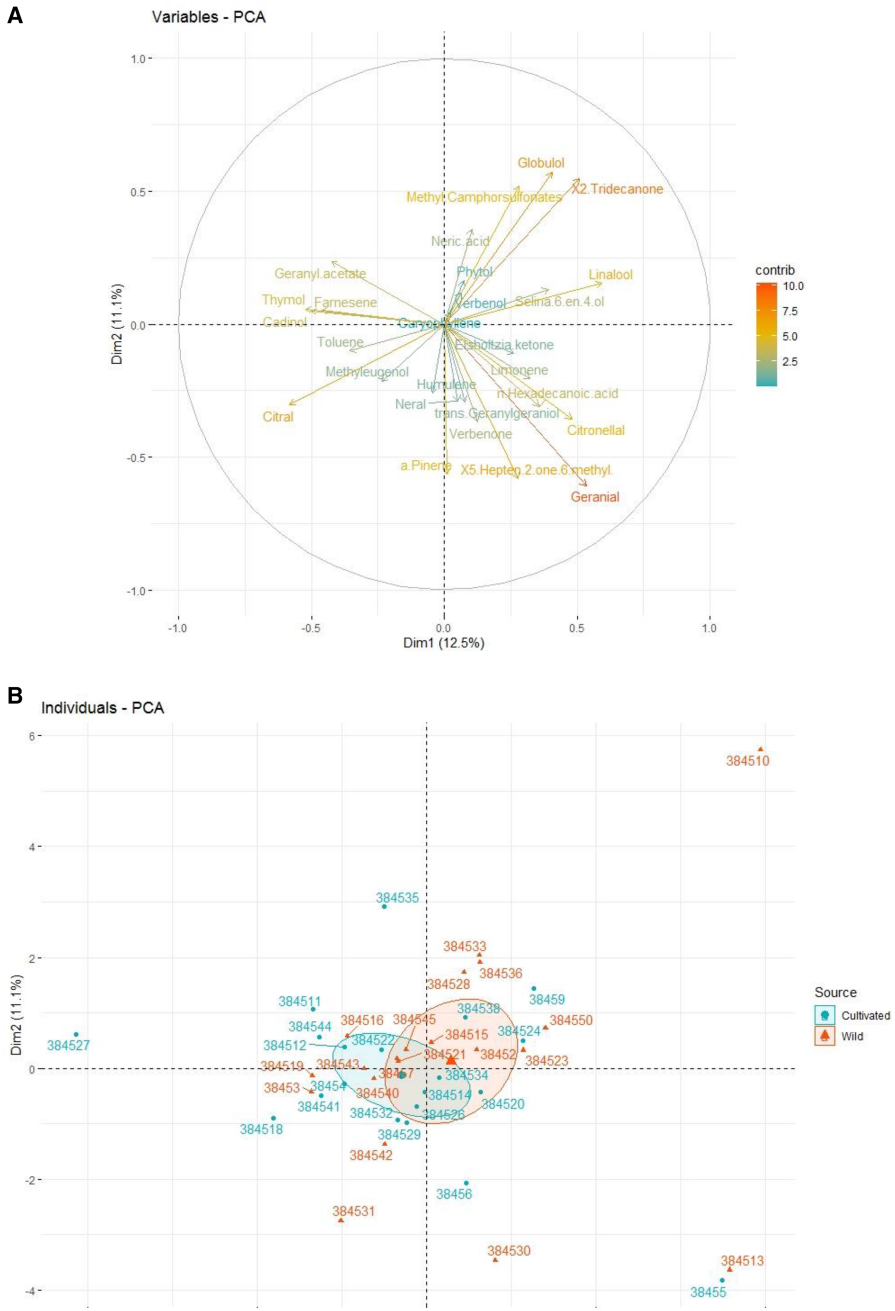
PCA of the chemical diversity of *C. citratus* based on EO components (A) and the contribution of each compound to the diversity of the considered accessions (B). The wild and cultivated accessions distinguished by different colors, *i.e*., blue for cultivated and red for wild.

The relationship between geographical areas and chemical diversity was revealed by the combination of principal component analysis Axes 1 and 2 (Fig. 2). The inertia of Axis 1 (12.5%) was close to that of Axis 2 (11.1%), and the sum of the two axes represented 23.6% of the total inertia variance. Taking geographical locations into account, the accessions 38454 (Dhaka), 384512 (Amritsar), and 38456 (Virgin Island) were phytochemically different from the Pakistani accessions based on the nature of the compounds and the citral concentration. The cultivated accessions of Pakistan 384527 (Hafizabad), 384535 (Karachi), 38455 (Shiekupura), 384518 (Faisalabad), and wild accessions 384510 (Kunjah), 384513 (Pattoki), 384530 (Karachi), 384531 (Karachi), 384542 (Daska) were positioned far from the axis and shown in a different cluster of PCA (Fig. 2). The Pakistani accessions 38452, 384515, and 384521 fall in the same cluster and are phytochemically close to each other. These three accessions have some common compounds, such as 5-hepten-2-one, 6-methyl, verbenol, and citral. On the other hand, three wild accessions, 38457, 384540 and 384543, were negatively correlated with each other in terms of different chemical compositions as well as the concentrations of compounds. As α-pinene was absent in accession 384540, while it was present at higher concentrations in accession 38457 (15.5%) than in accession 384543 (3.1%). With reference to neral, accession 384514 constituted a major contribution (12.58%), followed by 384522 (6.34), 384526 (1.34) and 384534 with no concentration.

### Region-specific compounds

Four compounds, (2,2-dimethyl-6-methylenecyclohexyl) butanal, Pentadeca-1,3,7,12,14-pentaen-7-ol-9, 3,7-cycloundecadien-1-ol,1,5,5,8-tetramethyl and 1,5,9-undecatriene-2,6,10-trimethyl, were detected only in Pakistani accessions 384518 (Faisalabad), 38452 (Alipurchatha), 384522 (Sargodha) and 384510 (Kujah), respectively, whereas two compounds, phenol, 2-(3,7-dimethylocta-2,6-dienyl), and 1-cyclohexyl-2-buten-1-ol, were specific to the United States (Table 3). One compound, 2-propenal,3(3,4 dimethoxyphenyl), was particularly related to the Bangladesh site, and pentadeca-1,3,7,12,14-pentaen-7-ol-9-one was specific to Indian accessions. All these compounds were found at concentrations greater than 1% in EOs.

**Table 3:** Region-specific compounds identified in essential oil of *C. citratus*.

Compound names	Accession no.
4-(2,2-Dimethyl-6-methylenecyclohexyl)butanal	384518
Pentadeca-1,3,7,12,14-pentaen-7-ol-9	38452
3,7-Cycloundecadien-1-ol,1,5,5,8-tetramethyl	384522
1,5,9-undecatriene-2,6,10-trimethyl	384510
Phenol, 2-(3,7-dimethylocta-2, 6-dienyl)	38456
1-Cyclohexyl-2-buten-1-ol	38456
2-Propenal,3(3,4 dimethoxyphenyl)	38454
Pentadeca-1, 3, 7, 12, 14-pentaen-7-ol-9	384512

### Percentage of total oil yield and citral concentration

**Figure 3:**
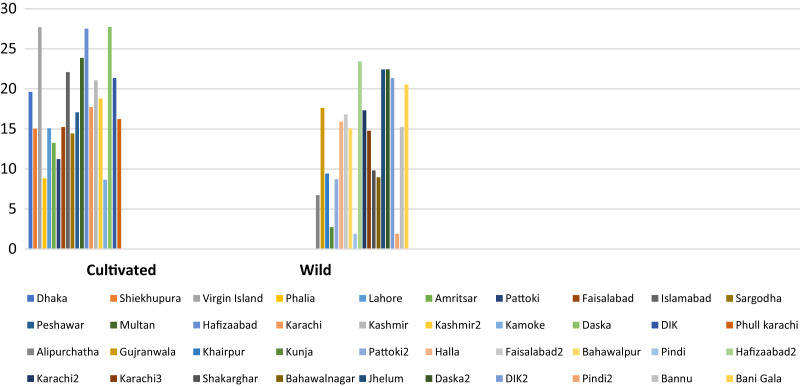
Comparison of citral content in cultivated and wild accessions of *C. citratus*.

The average oil yield among wild accessions was observed to be 1.17 ± 0.21 mL/g, whereas cultivated accessions showed 1.32 ± 0.42 mL/g, as presented in Table 3. The maximum oil yield was found in accession 384518 (Faisalabad), and the minimum was found in accession 384550 (Bannu). The citral percentage in all accessions is represented in Table 4. The comparison of citral percentage among accessions revealed that citral percentage was higher in cultivated accessions than wild accessions (Fig. 3).

**Table 4:** Essential oil yield and percentage area of citral from various accessions of *C. citratus*.

Accessions no	Location	Oil yield (ml)	Citral %Area in 1 µl	Accession no	Location	Oil yield (ml)	Citral %Area in 1 µl
38452	AlipurChatha	0.91	6.73	384524	Peshawar	0.42	7.06
38453	Gujranwalla	0.72	17.62	384526	Multan	0.99	23.87
38454	Dhaka	1.21	19.62	384527	Hafizabad	1.26	27.52
38455	Sheikhupura	0.82	12.04	384528	Hafizabad (JalalpurBhattian)	1.03	23.43
38456	USA (Virgin Island)	0.51	27.71	384529	Karachi University	1.28	17.72
38457	Khairpur	1.14	19.42	384530	Karachi	0.91	17.31
38459	Phalia	0.75	8.82	384531	Karachi	0.98	14.76
384510	Kunja	0.41	2.73	384532	Phull Karachi	1.12	16.21
384511	Model town Lahore	0.63	15.09	384533	Shakarghar	0.64	9.8
384512	Amritsar	0.54	13.27	384534	Kashmir	0.34	12.06
384513	Pattoki	0.51	8.71	384535	Kashmir	0.45	11.81
384514	Pattoki	0.86	11.23	384536	Bahawalnagar	0.96	8.96
384515	Halla	0.42	15.92	384538	Kamoki	0.66	8.65
384516	Manawala (Faisalabad)	0.65	16.79	384540	Jhelum	0.57	22.41
384518	Faisalabad	1.32	19.24	384541	Daska	1.26	27.74
384519	Bani Gala (Islamabad)	0.41	15.22	384542	Daska	1.09	22.45
384520	Islamabad	0.66	12.09	384543	Dera Ismail Khan	1.17	24.7
384521	Bahawalpur	0.56	15.09	384544	Dera Ismail Khan	1.21	21.31
384522	Sargodha	0.87	14.43	384545	Pindi	0.25	2.18
384523	Pindi	0.34	1.92	38450	Bannu	0.21	3.42

### Genetic diversity based on functional molecular markers between the wild and cultivated accessions of *C. citratus*

**Figure 4:**
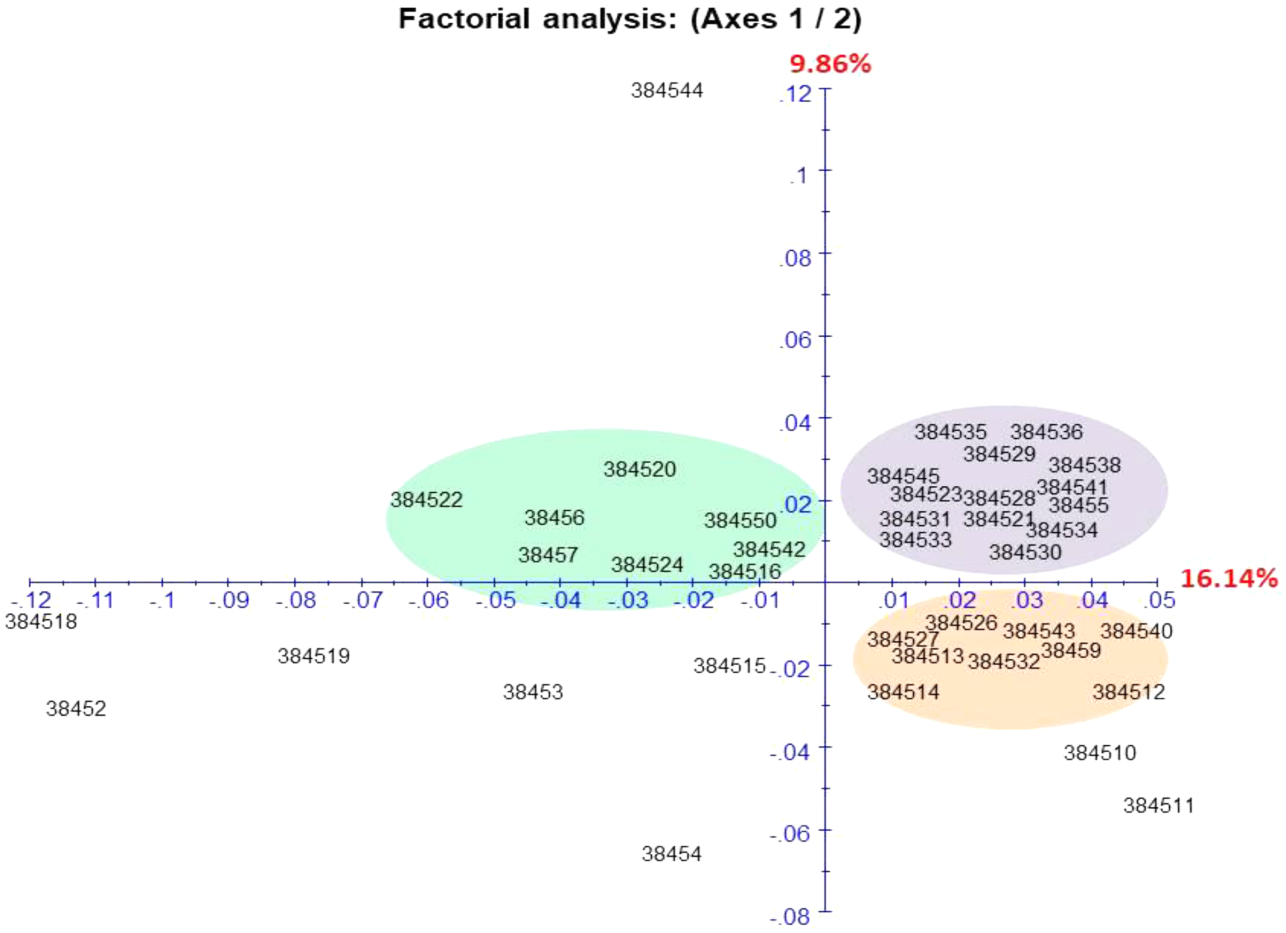
Factorial analysis (DFA) of the diversity of the *C. citratus* in wild and cultivated accession.

**Figure 5:**
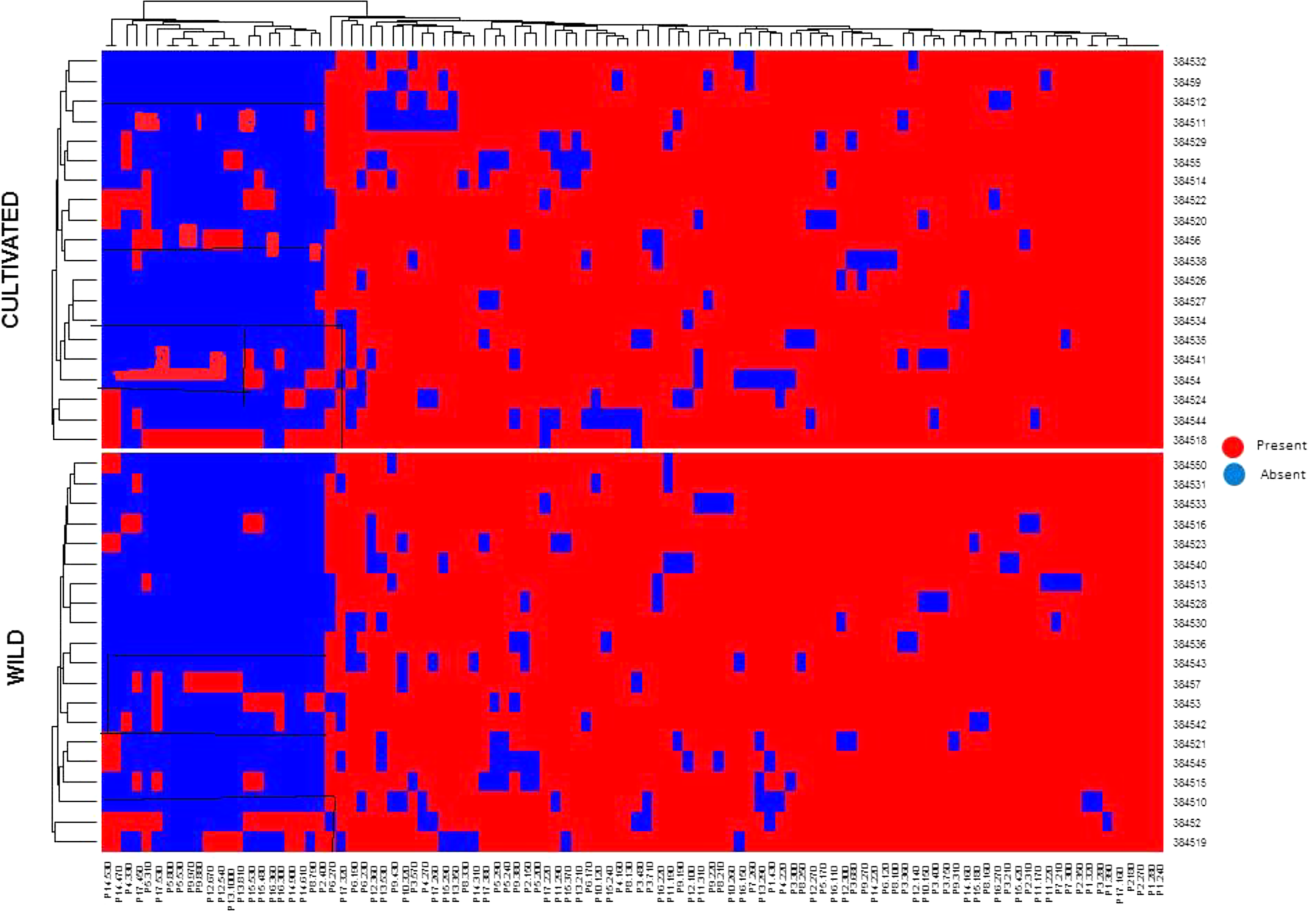
Heat map of genetic diversity and the relationship between 40 accessions of *C. citratus* based on binary data of DNA amplified with seventeen primers. Twenty cultivars (upper) and 20 wild (below) the presence of band represented by red and absence by blue color.

Seventeen functional molecular markers were chosen for assessing the genetic diversity of wild and cultivated accessions (Table 5). The selected functional molecular markers were based on polymorphism and the quality of bands. The number of alleles per primer ranged from five to 10. The PIC value for the functional molecular marker ranged from 0.48–0.83 (Table 5). Genotypic data were used to conduct a factorial analysis based on similarity and dissimilarity indices to characterize the diversity among all accessions of *C. citratus* (Fig. 4). The first two axes of the factorial analysis F-1 and F-2 showed maximum variability and represented 26% diversity (Fig. 4). These accessions belonged to different regions of Pakistan but showed less genetic diversity. Accessions 384516 and 384518 belonged to Faisalabad but were represented in different clusters and showed a negative correlation. The accessions 384527 and 384528 collected from Daska were represented in different blocks (384527 in brown and 384528 in gray block) in the factorial analysis (Fig. 4). The accessions 38452 (Alipurchatha), 384511 (Lahore), and 384544 (DIK) were also positioned separately from all other accessions. Site-specific grouping was observed. The accessions 38454 (Dhaka), 384512 (Amritsar), and 38456 (Virgin Island) were genetically different from accessions collected from Pakistan and displayed a negative correlation. The heatmap represents the genetic diversity between wild and cultivated accessions of *C. citratus*. In the cultivated accessions, 384518 (Faisalabad), 38454 (Dhaka), 384512 (Amritsar), 38456 (Virgin Island), 384520 (Islamabad), and 384514 (Pattoki) were found, while in the wild accessions, 384519 (Islamabad), 38452 (Alipurchatha), 384510 (Kunjah), 384515 (Halla), 384542 (Daska) and 38457 (Khairpur) were found to be genetically diverse and represented the polymorphism with the primers P3, P4, P5, P8, P14, P17, P12, P13, P15, and P16 (Fig. 5).

**Table 5:** Information on primer sequences, total number of bands and polymorphic bands detected in *C. citratus* genotypes using markers based on three gene families.

Sr.No	Primer sequences	Temp. (°C)	Marker size ranged (bp)	Number of bands	PIC
1	GCCAGAAGGAAAAGAGA GAAACGGGTCCAATGGA	58	430–240	5	0.48
2	CAACGGAGTTGATGGTA CTCTCAGCTTTGTCTGCA	58	400–150	8	0.33
3	CACATCCTATGGTGTGA GATCAGTGGAATGCCTGA	58	750–210	10	0.52
4	TGCAAGTGGAGATTGGA GTGTCAGGAATCCTCCAA	58	330–160	7	0.61
5	GCAAGTGGAGATTGGAA TGTGTCAGGAATCCTCCA	58	800–170	8	0.72
6	GCTCAGCGTGGTGTTGA TCCAATCTGCACTTGCA	58	270–160	5	0.50
7	TCCTTTGTCTCAGCTCA TCCACAGCTTTGTGA	58	390–210	4	0.47
8	GCAAATGCAAGTGGAGA AGGAATCCTCCAACGGA	58	790–100	7	0.62
9	GATGGTCTTCCGCGGTA CACTGGAAGGCGTGCA	57	970–190	8	0.72
10	CGGCTTGCTCATGGA GAGAAATAGGTGCGTGA	57	320–120	5	0.63
11	GACCCAAGCAACGTCA GTGGGTTATGGCCCACA	58	310–170	5	0.46
12	GACGTGCCACTCTGCA ACCCTAGGCTAAGGTGGA	58	670–130	5	0.56
13	CCACCTTGACGACCCAA TGGCCCACATATTCACCA	58	1,000–210	10	0.63
14	ACGTGCCACTCTGCAA ACCCTAGGCTAAGGTGGA	58	900–160	7	0.68
15	GGGCCATAACCCACGA ATTGGAGCCCCGGTGA	58	530–180	7	0.57
16	CCTGTACGACCCAAGCA TGGCCCACATATTCACCA	58	360–130	6	0.58
17	TTTAGTGCTGGTTGTCGC TGGGAAGTCCTCGTGTGTTGA	59	530–160	9	0.52

## Discussion

Many factors, such as the variety, time of cultivation, harvest condition, geographical location, plant preparation, and extraction method, affect the chemical composition of EOs (Tajidin et al., 2012). In the present study, all the accessions collected from different geographical zones showed variation in phytochemical and genetic traits. It was observed that the accessions collected from temperate regions showed a high oil yield (Table 3). This might be because high temperature increased photosynthesis and provided optimal conditions for the accumulation of EOs (Tawatsin et al., 2006). However, no relationship was found between precipitation and EO composition.

The method of extraction also influenced the oil yield. In the present research, the leaves of wild and cultivated accessions of *C. citratus* were harvested at a mature stage and dried under shade conditions for 72 h to obtain maximum oil yield and citral contents. In a previous study, the essential oil from the leaves of *Eucalyptus sargentii* was extracted from three different methods: shade drying, oven drying, and sun drying. Hanaa et al. (2012) concluded that shade drying is the best drying method rather than oven drying or sun drying. In the present study, the hydrodistillation method was used for the extraction of the essential oil of *C. citratus* because of its simplification and fastness with less heat generation compared to other methods. As in a previous study, the essential oil of basil was extracted by three different methods: steam distillation, microwave distillation, and hydrodistillation. The authors found that hydrodistillation is the best method for the extraction of essential oil because it prevents the loss of volatile compounds by generating less heat (Charles & Simon, 1990).

### Variation in the chemical composition of volatile compounds and oil yield in *C. citratus*

**Figure 6:**
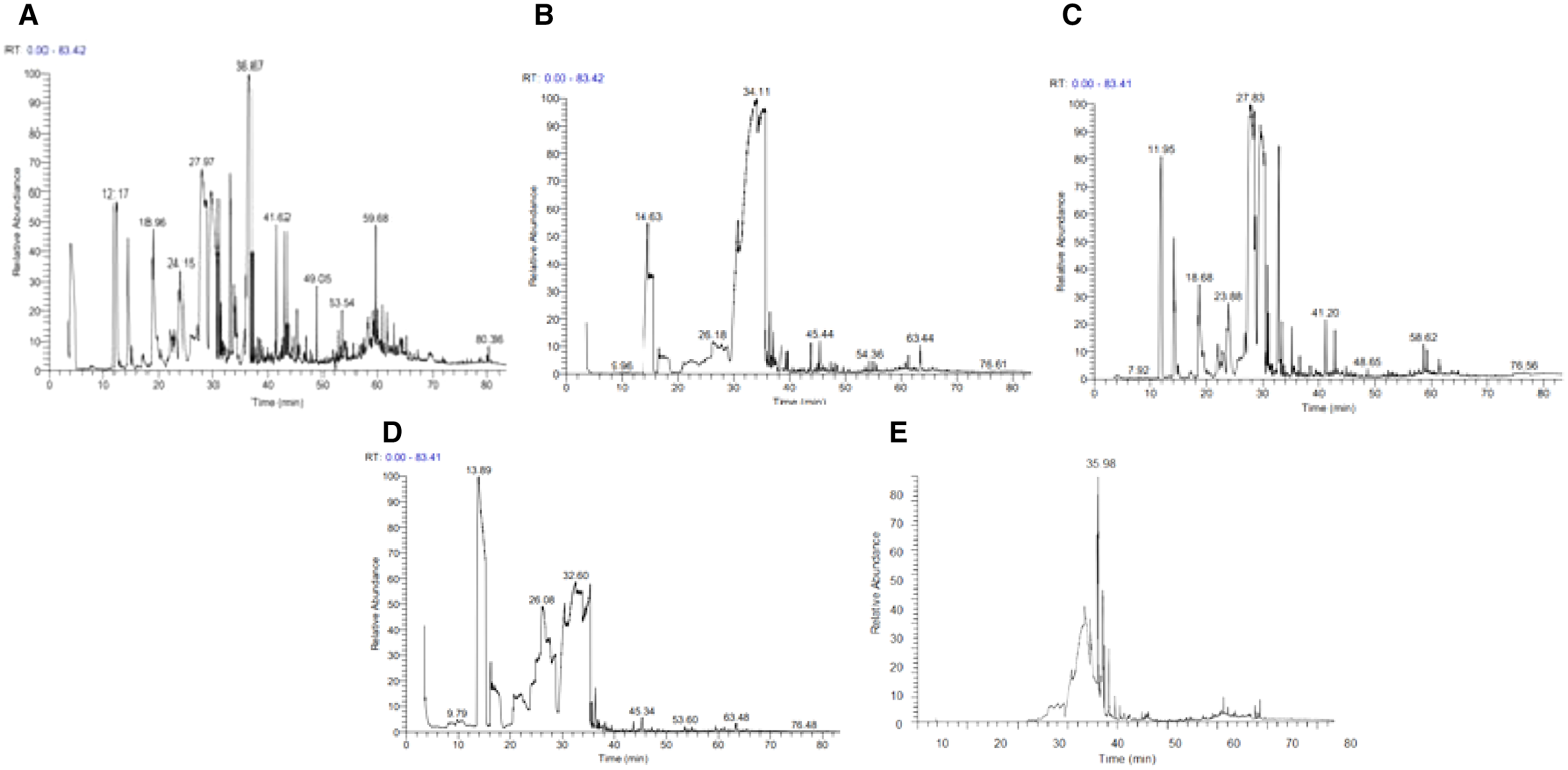
GC-MS chromatograph of essential oil components of *C. citratus* and citral standard. (A) 38453, (B) 38456, (C) 384519, (D) 38457, (E) citral standard.

There was significant variation in EO composition among the accessions collected from different geographical areas. Among all accessions, the dominant volatile component was citral, except for 384510, 384523, 384514, and 384530. These results were in accordance with the observations reported by Plata-Rueda et al. (2020) and Echeverrigaray et al. (2003), showing the highest citral content in *C. citratus and Cunila galioides*. In the present study, α-pinene was found in up to 15.57% of many accessions of *C. citratus*. In a previous study, a sufficient concentration of α-pinene was also detected in the EOs of *C. citratus* (Djerrad, Kadik & Djouahri, 2015). The difference in the concentration is probably due to differences in geographical origins. The accessions 384510 and 384523 were found to be rich in verbenol, 5-hepten-2-one, and 6-methyl as oil dominant components. Neral was found to be a major component in only two accessions, 384514 and 384530, with proportions of 12.58% and 21.31%, respectively. These results are in accordance with Luis et al. (2017), where neral (24.6%) was found to be the dominant component in *C. citratus* rather than citral (18.7%). In the present study, Methyleugenol was detected in a high proportion, *i.e*., 11.19% agreeing, with the findings of Shaikh, Suryawanshi & andMokat (2019), who found a higher amount of methyleugenol (13.09%) in *C. citratus*. Verbenone and limanone were present in more than 1%, whereas in the literature, they were reported up to 1% (Moumni et al., 2021). The difference in the concentration might be due to the difference in the number of accessions, as in the literature, only one accession was analyzed by GC–MS, while in the present study, 40 accessions were used (Fig. 6). The effect of the environment and geography on the EO composition was evident from the above results. Many researchers have proven that the variation in the chemical composition is due to geographical and environmental factors (Djerrad, Kadik & Djouahri, 2015). The essential oil yields are characterized as the first important indication of the difference between the wild and cultivated accessions of *C. citratus*. The obtained essential oil was light yellow with specific citrus essence. Cultivated accessions showed a higher oil yield (1.32 ± 0.42 mL) than wild accessions (1.17 ± 0.21 mL) (Table 3). In contrast to a previous study related to oat accessions, wild accessions yielded more oil than cultivated accessions (Leonova et al., 2008). However, in our findings, cultivated accessions had higher oil yields than wild accessions, supporting Julio et al. (2015), who found higher oil yields in cultivated accessions of *Artemisia absinthium* than in wild accessions. The citral percentage was also higher in cultivated accessions than in wild accessions (Fig. 2). The difference in the oil yield and citral percentage and EO composition might be due to the difference in the temperature, altitude, and cultivation practice because the accessions 384513 (wild) and 384514 (cultivated) collected from the same geographical area Pattoki possess different EO profiles and oil yields, *i.e*., 0.51 and 0.86 mL, respectively (Table 3). Similarly, four accessions, 384529, 384530, 384531, and 384532, were collected from Karachi, two cultivated with higher oil yield and two wild with relatively low yield, indicating that the cultivation process might improve the oil yield and composition.

On the other hand, the cultivated accessions 384534 and 384535 were collected from Kashmir possessing lower oil yield (Table 3), indicating that altitude might also affect oil yield. These results agreed with previous findings in which higher oil yields were found in accessions of *Thymbra spicata* var. *spicata* L. collected from a lower altitude (Kizil, 2010). The reason for the high essential oil yield in the accessions at low altitudes might be due to temperature differences. The temperature is higher at a lower altitude, resulting in higher photosynthesis and providing optimal conditions for the accumulation of essential oil (Pirbalouti, Hashemi & Ghahfarokhi, 2013).

### Some medicinally important region-specific compounds identified for the first time in *C. citratus*

Four compounds, 4-(2,2-dimethyl-6-methylenecyclohexyl) butanal, pentadeca-1,3,7,12,14-pentaen-7-ol-9, 3,7-cycloundecadien-1-ol,1,5,5,8-tetramethyl and 1,5,9-undecatriene-2,6,10-trimethyl, were detected only in Pakistani accessions 384518 (Faisalabad), 38452 (Alipurchatha), 384522 (Sargodha) and 384510 (Kujah), respectively. Previously, these compounds were detected in the essential oils of *Olax acuminate, Lupinus varius* L., *Cinnamon zeylanicum, and Melaleuca cajuputi* and are currently used to treat Alzheimer’s disease in human beings (Sadiq et al., 2018). These compounds are also reported to possess cytotoxicity against human cancer cells (AL-Qudah, 2013) and to treat human stomach disorders (Tawatsin et al., 2006). Therefore, these compounds could be used in food supplements to boost the immune system against stomach diseases. Two compounds, phenol, 2-(3,7-dimethylocta-2,6-dienyl), and 1-cyclohexyl-2-buten-1-ol, found in United States accession 38456 were previously reported in the EOs of *Manilkarabidentata* and *Cicutavirosa* to be effective for anti-inflammatory (Lidder & Sonnino, 2012) and antibacterial activities (Kumar et al., 2007). These compounds might be used in cosmetic creams to prevent skin inflammation. One compound, Pentadeca-1,3,7,12,14-pentaen-7-ol-9-one, was specific to Indian accession 384512. This essential oil of *Lupinus varius* L. was reported to be effective in the treatment of human cancer cells (Riaz et al., 2018). Compound 2-propenal, 3 (3,4 dimethoxyphenyl), was detected only in Bangladesh accession 38454. Previously, it was detected in the EOs of *Echinophora tenuifolia* L. and used to treat kidney and digestive diseases (Babatunde et al., 2019). These compounds might be used in food supplements to provide strength to digestive systems.

### Compounds identified for the first time in the essential oil of *C. citratus*

The discovery of novel compounds has a great impact on the biochemical profiling of MAPs. Almost 30 compounds were reported from different accessions of *C. citratus* (Table 6). These compounds are organic in nature, constitute 0.5% to 0.95% and are reported for the first time in the essential oil of any plant. There is not sufficient literature available about their nature and medicinal potential; therefore, more research is required to explore their medicinal importance against fungal, bacterial, and viral infections.

**Table 6:** Novel compounds found in the essential oil of *C. citrate a*ccessions.

S. No	Compounds name	Accession numbers
1	4-Hexenoicacid,6-(acetyloxy)-4-methyl	384519, 384533
2	Benzoic acid, 4-(methylthio)	38452, 384529, 384530, 384533
3	2-Pentyne	384519
4	2-Norbornanol, 1,2-dimethyl	38456
5	9-Phosphabicyclo-[3.3.1]nonane	38456
6	1,3-Benzodioxole, 3a,7a-dihydro-2,2,4-trimethyl	38456
7	Cyclohexanone, 2,5-dimethyl-2-(1-methylethenyl)	384515
8	2-(1-Hydroxyethyl)-hydroxymethylbenzene	384511
9	Cyclobutaneethanol, a-methylene	384550
10	4-Imidazoleacetic acid, butyl ester	38457
11	3-Furancarboxylic acid, 2,4-dimethyl-, ethyl ester	38457
12	2-Butenoic acid, 2-methoxy-, methyl ester	384535
13	3-[2-(4-Methylphenylthio) ethyl]-4-H-sy dnone	384535
14	6-Methyl-6-nitro heptan-2-one	384535
15	1,2,2-(trimethyl-3-cyclopenten-1-yl)acetaldehyde	384535
16	1-Pentyne, 3-methyl-3-(1-methylethoxy)	384535
17	(2,4,6-Trimethylcyclohexyl) methanol	384535
18	3,3,5-Trimethylcyclohexyl acrylate	384534
19	Cyclobutaneethanol, a-methylen	384529
20	3-ethenyl-2-ethoxypyrazine	38457, 384543, 384544
21	N-(Dimethyl ThioPhosphinyl)ethylamine	384521
22	2-Pentyne, 4,4-dimethyl	38457
23	Cyclohexene, 3-(3-methyl-1-butenyl)	384531
24	2,3,5-Trimethylanizole	384529
25	Ethyl 1-acetonyl-2-oxocyclopentanecarboxylate	384535,384538
26	5-Methoxy-[1,2,3]oxadiazole	38457, 384528, 384535, 384538
27	Benzene ethanethioic acid, S-methyl ester	384528, 384538
28	Cyclohexanone,2,5-dimethyl 2-(1-methylethenyl)	38457, 384526, 384527, 384531, 384532, 384534, 384541, 384542
29	3,7-cyclo undecadien-1-ol-1, 5, 5, 8-tetramethyl	384522
30	Cyclopropane Carboxaldehyde, 2-methyl, 2-(4-methyl-3-pentenyl)trans	38455

### Organization of the genetic diversity of *C. citratus*

Essential oil composition is important for food production and is used as a natural preservative (Musso et al., 2017). However, the chemical composition of essential oil is not stable because of exogenous and endogenous variations. Oil composition can provide extensive information but is limited to varietal classification. Therefore, a molecular study is necessary to confirm the status of the variety. Functional molecular markers offer an exclusive dimension to classify accessions irrespective of environmental conditions and plant growth (Amom & Nongdam 2017). The constructed heatmap encoding genetic information of wild and cultivated accessions showed high polymorphism with primers P3, P4, P5, P8, P12, P13, P14, P15, P16, and P17. However, higher polymorphism in cultivated accessions has suggested that cultivated accessions are genetically more diverse. These findings agreed with previous findings of grapevines, in which cultivated accessions showed more genetic diversity than wild accessions (Riaz et al., 2018). In this study, the significant PIC value was given by primers P3, P4, P5, P8, P10 P14, P17, P12, P13, P15, and P16 (Table 2), except for P2, which showed a low PIC value. The present findings agreed with the literature, where these primers were used to amplify other members of the genus *Cymbopogon* to reveal high polymorphism (Kumar et al., 2007). For further evaluation, factorial analysis (FA) was used to transfigure a large dataset in a concise manner (Fernández-Sestelo & Carrillo, 2020). Many scientists have used this technique for the comprehensive evaluation of the genetic diversity of their desired crops (Kumar Ganesan et al., 2014). Site-specific grouping was observed for genetic diversity in accessions 38454 (Dhaka), 384512 (Amritsar), and 38456 (Virgin Island). Pakistani accessions with different geographical origins also showed diversity among them. Genetic diversity might arise due to environmental and geographical variability, and various studies have revealed the effect of environmental and geographical factors on the genotype (Kumar Ganesan et al., 2014). Two accessions, 384516 (wild) and 384518 (cultivated), collected from Faisalabad presented different genetic behaviors; similarly, two accessions, 384519 (wild) and 384520 (cultivated), collected from Islamabad presented different components, suggesting that cultivation and geographical location also affected the plant genotype (Fig. 5). The present findings are in accordance with previous reported research regarding the genetic diversity of wild and cultivated cucumber, where the majority of upregulated genes in cultivated cucumber improved taste during domestication (Abdel-Salam et al., 2020).

### Correlation between phytochemical and molecular traits

A positive correlation was observed between the phytochemical and molecular traits of *C. citratus*. The accessions were found to be phytochemically and genetically diverse and strongly associated with geographical origin. A positive correlation was observed between the EO yields with altitude and temperature, but a poor correlation was observed with precipitation, suggesting no effect on the composition of the essential oil of *C. citratus*.

## Conclusion

The current study evaluated the diversity in the essential oil chemical profile and genetic makeup among wild and cultivated accessions of *C. citratus* collected from four countries. Molecular data showed a strong relationship with the essential oils of *C. citratus*. The essential oil yield showed a significant association with geographical origin, altitude, and domestication, suggesting an edit effect on the biosynthesis of EOs. Moreover, molecular analysis using functional molecular markers revealed that the accessions of *C. citratus* were highly polymorphic and genetically diverse in association with their geographical origin. Phytochemical and genetic variation was more pronounced in the cultivated accessions than in the wild accessions. These results lay the foundation for breeding and genetic enrichment programs of *C. citratus*. However, more research is required to improve the knowledge about domestication and genetic variability among wild and cultivated accessions. Further analyses of EOs are necessary to evaluate medicinally important novel compounds for their utilization in the pharmaceutical and cosmetic industries.

## Supplemental Information

10.7717/peerj.13505/supp-1 Supplemental Information 1 Raw Data set of Identified Oil compounds.

10.7717/peerj.13505/supp-2 Supplemental Information 2 Raw data set of amplified primers.

10.7717/peerj.13505/supp-3 Supplemental Information 3 R script for Heat maps.

## Additional Information and Declarations

### Data Availability

The following information was supplied regarding data availability: The raw data are available in the Supplemental Files.
